# Lipid metabolic reprogramming in clear cell renal cell carcinoma: focus on high-density lipoprotein-related pathways

**DOI:** 10.3389/or.2026.1887460

**Published:** 2026-09-01

**Authors:** Lize Wu, Huaying Xue, Yifei Wu, Ningning Shen, Zhiqing Yang, Lifang Gao, Wenxia Ma, Chen Wang

**Affiliations:** 1 Department of Pathology, Second Clinical Medical College of Shanxi Medical University, Taiyuan, China; 2 Department of Pathology, Basic Medical College of Shanxi Medical University, Taiyuan, China; 3 Department of Pathology, the Second Hospital of Shanxi Medical University, Taiyuan, China

**Keywords:** cholesterol efflux, cholesteryl ester, clear cell renal cell carcinoma, high-density lipoprotein, lipid droplet, lipid metabolic reprogramming, SCARB1, SOAT1

## Abstract

Clear cell renal cell carcinoma (ccRCC) is one of the most prevalent solid tumors characterized by lipid metabolic reprogramming, with extensive lipid droplet accumulation in tumor cells as a hallmark feature. Traditionally, this phenotypic trait is attributed to enhanced fatty acid synthesis, increased triglyceride (TG) storage, and impaired β-oxidation. However, emerging transcriptomic and lipidomic evidence suggests that cholesterol metabolism is also profoundly reprogrammed in ccRCC, particularly via increased reliance on high-density lipoprotein (HDL)-derived cholesterol. The classical fatty acid-TG pathway and the HDL-cholesterol axis are metabolically linked through shared substrates, collectively shaping lipid homeostasis in ccRCC. In this review, we summarize recent advances in ccRCC lipid metabolic reprogramming, with a focus on HDL-related cholesterol metabolism. We discuss the mechanisms involving von Hippel-Lindau (VHL) loss and hypoxia-inducible factor (HIF) activation, including SCARB1-mediated cholesterol uptake, LXRα-ABCA1/ABCG1-dependent cholesterol efflux, and SOAT1-driven re-esterification of free cholesterol into cholesteryl esters (CE). Furthermore, we outline potential therapeutic targets within these pathways and highlight critical knowledge gaps that merit further investigation. This review provides a conceptual framework for understanding HDL-centered cholesterol metabolism in ccRCC and highlights potential metabolic vulnerabilities for therapeutic targeting.

## 1 Introduction

Clear cell renal cell carcinoma (ccRCC) is the most common type of renal cell carcinoma, accounting for approximately 75%–80% of all cases (1). Its defining morphological feature is the optically clear cytoplasm of tumor cells, which largely reflects the abnormal accumulation of neutral lipids and glycogen within the cytoplasm (2, 3). Accumulating evidence from molecular pathology and lipidomics indicates that this clear-cell phenotype is not merely a histological manifestation, but rather the phenotypic expression of tumor-specific metabolic reprogramming (2, 4). In multiple tumors, for instance colorectal cancer, lipid metabolism has been reported to be a necessary condition for the growth of tumor cells, the storage of lipids in cytoplasmic lipid droplets not only provides substrates for membrane biogenesis, but also contributes to the buffering of oxidative stress, the maintenance of endoplasmic reticulum (ER) homeostasis, and remodeling of the tumor microenvironment, thereby supporting tumor cell survival and adaptation. However, excessive lipid accumulation or dysregulated lipid handling may increase susceptibility to lipid peroxidation and oxidative damage, creating potential vulnerabilities that can be therapeutically exploited (5–9).

Development of ccRCCs is closely related with mutation or dysregulation of the von Hippel-Lindau (VHL) gene (1). Loss of VHL function places tumor cells in a pseudohypoxic state and results in the persistent activation of hypoxia-inducible factor (HIF) signaling (1, 10). Among HIF family members, HIF-2α is more stably and persistently activated in ccRCC and is widely recognized as a central driver of the pseudohypoxic program and its associated metabolic alterations (5, 10). Aberrant activation of the VHL-HIF axis affects not only cellular energy metabolism, but also lipid synthesis, oxidative utilization, and storage, ultimately yielding a metabolic phenotype characterized by prominent lipid droplet accumulation (2, 5, 11). Historically, lipid dysregulation in ccRCC has been primarily explained by enhanced fatty acid (FA) synthesis, increased triglyceride (TG) production, and the suppression of FA β-oxidation (11, 12). This classical framework accounts for part of the mechanism underlying lipid droplet expansion and the emergence of the clear-cell phenotype (2, 11).

However, accumulating evidence suggests that lipid dysregulation in ccRCC extends beyond the conventional fatty acid-TG pathway, with cholesterol metabolism also playing a significant role. For instance, Simeth et al. reported a marked enrichment of cholesteryl esters (CE) in ccRCC tissue (13), whereas Riscal et al. demonstrated that key enzymes involved in de novo cholesterol synthesis are not substantially upregulated, suggesting a greater reliance on exogenous cholesterol acquisition (14). In ccRCC, the expression of scavenger receptor class B type 1 (SCARB1) is consistently elevated and is closely associated with the selective uptake of high-density lipoprotein (HDL)-derived cholesteryl esters (7, 15). Collectively, these observations indicate that, beyond the classical FA-TG framework, HDL-mediated cholesterol uptake may represent another major pathway contributing to lipid droplet accumulation and lipid metabolic reprogramming in ccRCC (14–16).

In this review, we summarize current advances in lipid metabolic reprogramming in ccRCC, including the classical fatty acid-TG pathway as well as HDL-related cholesterol influx, insufficient cholesterol efflux, and cholesterol esterification. We further discuss the molecular mechanisms through which these two pathways coordinately regulate lipid metabolism in ccRCC. Finally, we outline potentially targetable nodes within these pathways and review current progress in this area, with the goal of improving our understanding of the distinctive morphology of ccRCC, clarifying the metabolic basis of lipid accumulation, and highlighting current challenges and clinical prospects in this field.

## 2 Methods

### 2.1 Literature search

The literature search was conducted based on PubMed, Web of Science Core Collection, and China National Knowledge Infrastructure (CNKI), covering publications from database inception to January 2026. The search aimed to identify relevant studies on lipid metabolic reprogramming in clear cell renal cell carcinoma, including its uptake, efflux, esterification and stabilization. Search terms included “clear cell renal cell carcinoma”, “high-density lipoprotein”, “cholesteryl esters”, “SCARB1”, “SOAT1”, “lipid metabolic reprogramming”, “metabolic reprogramming,” and “tumor metabolism”. Following the primary screening of the literature, the reference lists of relevant articles were further manually screened to be included into further studies.

### 2.2 Literature data collection and analysis

Studies were selected based on their relevance to the topic, with particular emphasis on research investigating molecular mechanisms, metabolic pathways, and tumor progression associated with lipid metabolism in ccRCC. Priority was given to the peer reviewed original research, reviews, and translational studies. The selected researches were then classified into different subgroups based on their topics. The analysis is structured around three key themes: the uptake, efflux or esterification of fatty acid and cholesterol.

### 2.3 Ethical statement

This review is based on previously published researches and does not involve any new human or animal experiments, ethical approval and informed consent are not required.

## 3 The classical FA-TG lipid metabolic pathway in ccRCC

CcRCC is characterized by the typical appearance of cytoplasmic clearing (2, 17). Previous studies have largely attributed this phenotype to loss of the VHL gene, which induces persistent HIF activation, thereby enhancing fatty acid synthesis and suppressing lipid β-oxidation, and ultimately driving lipid metabolic reprogramming in tumor cells (2, 5, 11, 17). This classical metabolic pathway provides an important basis for understanding the clear-cell phenotype of ccRCC (2, 11, 17).

### 3.1 Lipid synthesis centered on SREBPs

The sterol regulatory element-binding protein (SREBP) family serves as the core transcriptional regulators of lipid synthesis in ccRCC cells (18, 19). Upon activation, SREBPs translocate into the nucleus and induce the expression of a series of downstream target genes (18, 19). Among these, SREBP1 primarily drives the expression of genes involved in fatty acid and TG synthesis, whereas SREBP2 mainly regulates key enzymes in cholesterol synthesis and the mevalonate pathway (18, 19).

In ccRCC, SREBP activation is regulated by multiple signaling pathways (2, 12, 20, 21). In particular, following second-hit inactivation of the VHL gene, sustained HIF activation can be induced (2, 17). It has been reported that HIF-2α upregulates the amino acid transporter SLC7A5 and enhances mTORC1 activity in VHL-deficient renal carcinoma cells; meanwhile, mTORC1 is known to regulate the subcellular localization of Lipin1, thereby promoting SREBP maturation and nuclear activity (20, 21). In addition, activation of mTORC1 can, through feedback mechanisms, promote the translation of HIF-1α mRNA and further increase HIF activity (22). Thus, the VHL-HIF signaling axis and the PI3K-AKT-mTORC1 pathway may cooperate to promote SREBP-mediated lipogenesis (2, 20–22).

It should also be noted that PI3K-AKT-mTORC1 is not the only route through which VHL-HIF signaling exerts its effects. Some studies have reported that HIF-2α may also activate SREBP function through upregulation of MED15, a subunit of the Mediator complex, thereby inducing lipid synthesis (12). The convergence of multiple oncogenic pathways forms a complex regulatory network that controls lipid synthesis in ccRCC, such that inhibition of a single pathway is unlikely to fully block intracellular lipid production (2, 3, 12).

### 3.2 PLIN2-mediated stabilization of lipid droplets

When lipid synthetic flux is markedly elevated, tumor cells must store lipids in a stable form, and lipid droplets provide the structural basis for this process (23, 24). Perilipin 2 (PLIN2) is a lipid droplet-coating protein that helps stabilize lipid droplets and restricts lipase access to the neutral lipid core, thereby maintaining droplet integrity and limiting premature lipid hydrolysis (23, 24). In ccRCC, sustained activation of HIF-2α has been shown to upregulate PLIN2 expression (5). Conversely, PLIN2 inhibition significantly exacerbates ER stress and reduces the viability of ccRCC cells (5). More recent studies and reviews further support that PLIN2 is closely associated with lipid droplet accumulation and tumor progression in renal cell carcinoma, reinforcing its role as an important executor of lipid storage remodeling downstream of oncogenic signaling (25, 26).

### 3.3 Impaired lipid “consumption” due to restricted fatty acid β-oxidation

In addition to increased synthesis, reduced lipid utilization also contributes to lipid accumulation (11, 27, 28). Carnitine palmitoyltransferase 1 A (CPT1A), the rate-limiting enzyme in fatty acid oxidation, determines whether long-chain fatty acids can enter mitochondria and undergo β-oxidation (11, 27). Previous studies have demonstrated that HIF-1α and HIF-2α can bind to the CPT1A promoter region and suppress its expression under hypoxic conditions in ccRCC (11). Downregulation of CPT1A reduces the flux of fatty acids into mitochondria, thereby limiting mitochondrial fatty acid oxidation and promoting intracellular lipid retention and accumulation (11, 27, 28). When free fatty acids accumulate rapidly and excessively within cells, they can induce lipotoxicity and ER stress (5, 29, 30). Under such conditions, excess lipids are generally redirected toward neutral lipid synthesis and subsequently sequestered into lipid droplets as a buffering mechanism against metabolic stress (5, 29–31).

### 3.4 Triglyceride synthesis and lipid droplet formation

**FIGURE 1:**
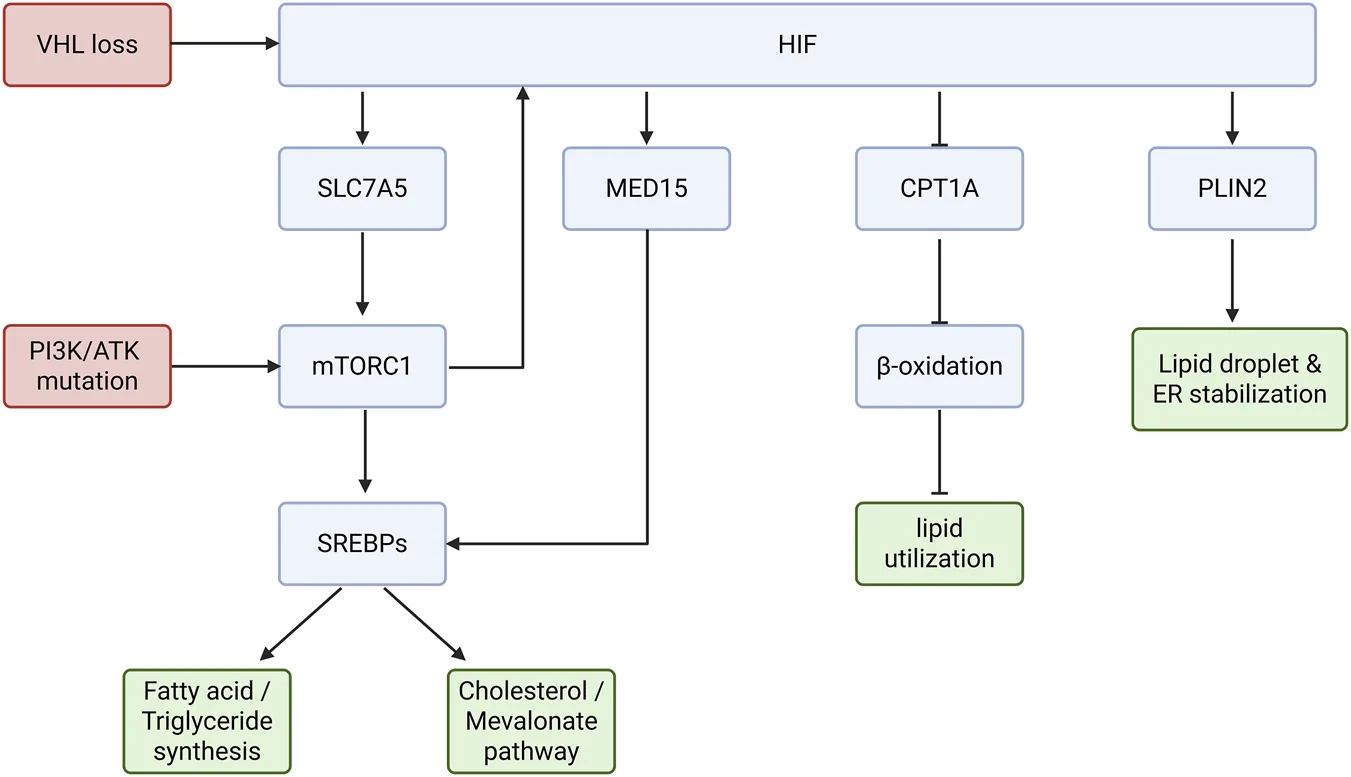
Classical lipid metabolic reprogramming in ccRCC. VHL loss in ccRCC sustains HIF-1α/HIF-2α activation, which promotes the SREBP-driven lipogenesis via SLC7A5-mTORC1 and MED15 signaling pathways, suppressing CPT1A and fatty acid β-oxidation, thus up-regulating PLIN2 to stabilize lipid droplets and maintaining ER homeostasis, resulting in lipid accumulation and the clear-cell phenotype in ccRCC. Created in BioRender. https://BioRender.com/bexxr4q.

Importantly, fatty acid synthesis is not equivalent to lipid droplet formation (31). Fatty acids must first be activated to fatty acyl-CoA, which is then converted into neutral lipids and stored in the form of lipid droplets (31). Diacylglycerol acyltransferases DGAT1 and DGAT2 are the key enzymes catalyzing the terminal step of TG synthesis (32, 33). Studies have shown that, in ccRCC, the JMJD6 gene can affect DGAT1 expression and thereby regulate lipid droplet formation and tumor cell proliferation (34). These findings support DGAT as an important regulator of lipid droplet biogenesis (Figure 1) (32, 34).

Taken together, sustained HIF activation resulting from VHL loss occupies a central position in lipid metabolic reprogramming in ccRCC (2, 3, 11, 35). On the one hand, persistent HIF activation promotes SREBP-associated lipogenic programs and increases fatty acid synthesis (11, 12, 20, 21). On the other hand, it stabilizes lipid droplet formation through structural proteins such as PLIN2 (5, 23). In addition, HIF suppresses CPT1A expression and restricts fatty acid entry into mitochondria for β-oxidation (11, 27). These changes collectively establish a TG metabolic profile in ccRCC characterized by enhanced synthesis, increased storage, and reduced consumption, thereby contributing to the characteristic clear-cell morphology (2, 5, 11, 27, 35).

## 4 The HDL-CE lipid metabolic pathway in ccRCC

CcRCC is characterized by a typical clear cytoplasmic phenotype (2, 17). Increasing evidence indicates that, in addition to the classical fatty acid-TG lipid metabolic pathway, HDL-derived cholesterol metabolism is also markedly remodeled in ccRCC (3, 14–16). Mass spectrometry-based studies have confirmed that both CEs and TGs are significantly increased in ccRCC tissues compared with normal tissues, with CE showing a particularly prominent increase (4, 15, 16, 36). Analysis of fatty acyl chain composition has further shown that normal renal cortex is enriched mainly in the essential fatty acid linoleic acid (C18:2), whereas ccRCC tissues display a marked increase in the non-essential fatty acid oleic acid (C18:1) (4, 13, 36). In ccRCC, this shift is reflected by enrichment of cholesteryl oleate (CE 18:1), in contrast to the predominance of cholesteryl linoleate (CE 18:2) in normal kidney tissue (13, 36).

Based on their chemical activity within membrane systems, cholesterol in cells can be considered as existing in different functional pools, including a more accessible fraction and a more sequestered membrane-associated fraction (37, 38). Importantly, this distinction mainly applies to FC within biological membranes rather than to CEs (37, 38). By contrast, CE represents the esterified storage form of cholesterol, which is generated to reduce excess free cholesterol and is mainly stored in lipid droplets or packaged into lipoproteins (16, 39). Lipid droplets enriched in neutral lipids, including CE and TG, are composed of a hydrophobic neutral lipid core surrounded by a phospholipid monolayer and coated by specific lipid droplet-associated proteins, which together help stabilize the droplet surface and regulate lipase access to the lipid core (23, 31, 40, 41).

### 4.1 Enhanced SCARB1-mediated HDL-CE uptake

#### 4.1.1 Association of VHL-HIF signaling with SREBP and SCARB1 expression

The double-hit inactivation of the VHL gene is the core driver event in ccRCC. Loss of VHL leads to persistent activation of HIF signaling, particularly HIF-2α, and thereby reshapes multiple metabolic programs involved in lipid storage and tumor progression in ccRCC (5, 10, 11, 42). Transcriptomic and metabolic studies further suggest that, compared with normal kidney tissue, ccRCC does not exhibit a coordinated enhancement of endogenous de novo cholesterol biosynthesis; instead, expression of several cholesterol synthesis-related genes is not consistently increased and may even be relatively reduced, whereas pathways involved in exogenous lipid/cholesterol acquisition are more prominent (2, 3, 10, 14, 28). This pattern is consistent with the findings of Riscal and colleagues, who showed that ccRCC displays cholesterol auxotrophy and depends, at least in part, on extracellular cholesterol supply mediated by SCARB1 (14).

Previous studies have shown that the SREBP family can bind SRE-like cis-elements within the SCARB1 promoter and enhance its transcription, providing a mechanistic basis for SREBP-dependent regulation of SCARB1 expression (18, 43). In parallel, PI3K-AKT-mTORC1 signaling is a well-established upstream regulator of SREBP activation, and HIF-2α can also intersect with mTORC1-related signaling in ccRCC (12, 20–22). Therefore, existing evidence supports the view that VHL loss and sustained HIF activation may promote cholesterol metabolic reprogramming partly through SREBP-associated transcriptional programs, which could contribute to increased SCARB1 expression and function (12, 20–22). However, the currently available evidence remains insufficient to conclude that HIF-2α directly binds the SCARB1 promoter and transcriptionally activates SCARB1 in ccRCC. Thus, the more rigorous interpretation is that the VHL-HIF axis is associated with, and may indirectly promote, SCARB1 upregulation through cooperative signaling networks involving SREBP, rather than representing a definitively established direct HIF-2α-to-SCARB1 transcriptional regulatory pathway (10, 12, 14, 42).

In addition, recent studies suggest that circRNAs may participate in SCARB1-related regulation in ccRCC. For example, circRNA-mediated stabilization or derepression of SCARB1 mRNA has been reported and may contribute to cholesterol metabolic remodeling and malignant progression (7, 44). Nevertheless, this line of evidence is still emerging, and whether circRNA-based regulation constitutes a broadly applicable epigenetic/post-transcriptional mechanism controlling the SCARB1-dependent HDL-CE uptake axis in ccRCC requires further validation (7, 44).

#### 4.1.2 SCARB1-mediated enhancement of HDL-CE uptake in ccRCC

**FIGURE 2:**
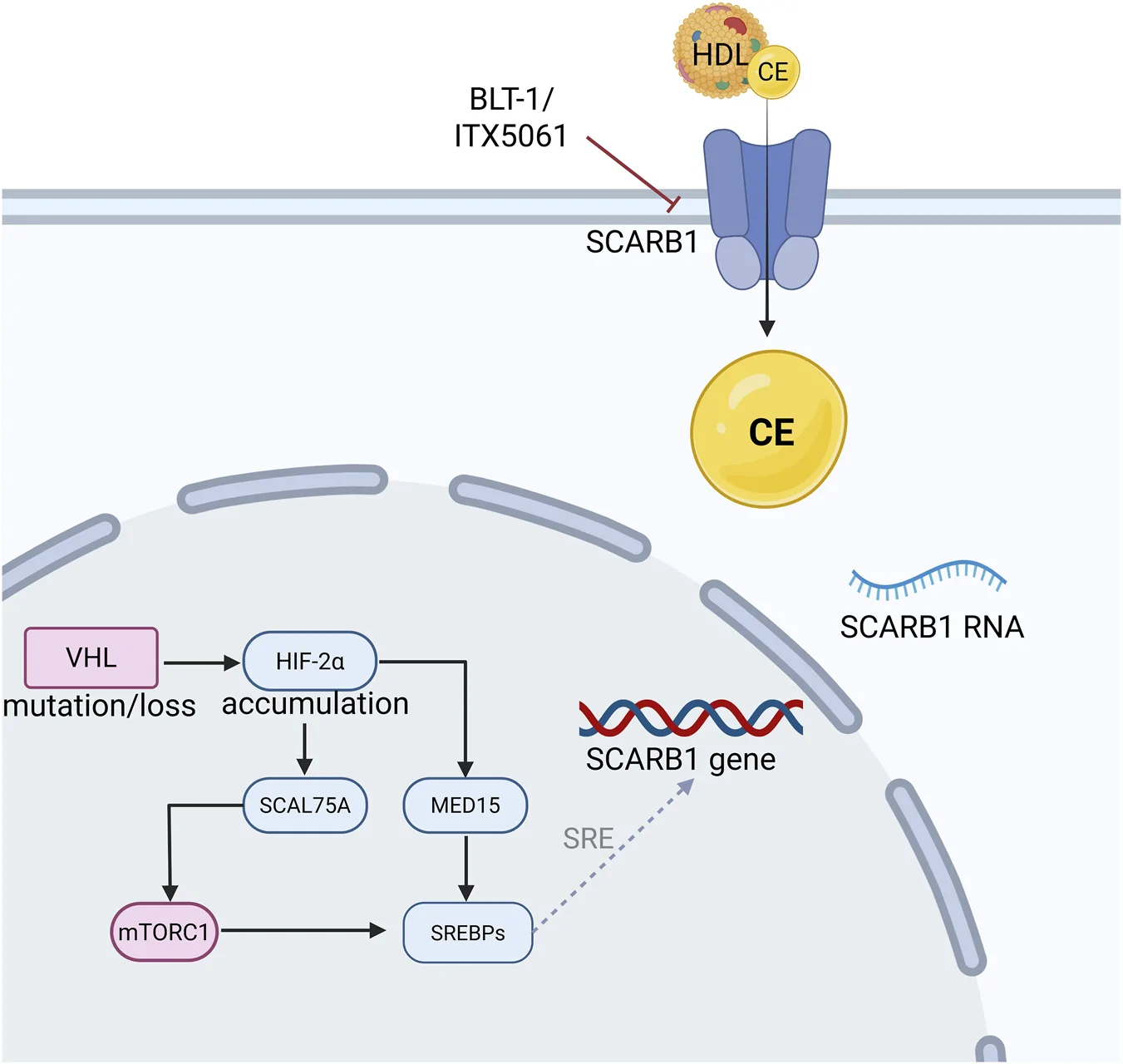
Proposed regulation of SCARB1 expression and HDL-derived CE uptake in ccRCC. VHL loss in ccRCC promotes HIF-2α accumulation, which enhances SCARB1 expression through SLC7A5–mTORC1, MED15, and SREBP associated signaling pathways. Increased SCARB1 expression supports selective uptake of HDL-derived cholesteryl esters. Although direct transcriptional regulation of SCARB1 by HIF-2α or SREBP remains unconfirmed, BLT-1 and ITX5061 are shown as potential inhibitors of SCARB1-mediated cholesterol uptake. Created in BioRender. https://BioRender.com/hchx49y.

Cholesterol uptake mainly occurs through two routes: SCARB1-mediated HDL uptake and LDLR-mediated LDL uptake. These two pathways differ mechanistically. LDLR internalizes the entire lipoprotein particle through endocytosis and subsequently delivers it to lysosomes for degradation, whereas SCARB1 mediates the selective uptake of CE from HDL without complete particle internalization (39) (Figure 2). This distinction is particularly relevant in tumors with increased dependence on exogenous cholesterol supply.

A substantial body of evidence from patient cohorts, transcriptomic analyses, and cell-based studies indicates that SCARB1 is significantly upregulated in ccRCC at both the mRNA and protein levels, whereas LDLR does not show a comparably consistent increase (15, 28). These findings suggest that, relative to LDLR-mediated LDL uptake, cholesterol acquisition in ccRCC may rely more heavily on the HDL-SCARB1 axis (10, 14, 15, 28).

Functional studies further support this view. In ccRCC cell lines such as 786-O, A498, and Caki-1, lipid-rich culture conditions or HDL supplementation increase intracellular CE accumulation and promote lipid droplet formation, whereas genetic suppression of SCARB1 or pharmacologic blockade with BLT-1 reduces HDL uptake, inhibits proliferation, induces G1-phase arrest, and increases apoptosis (15). Consistently, inhibition of SCARB1 also suppresses tumor growth in xenograft models, supporting a functional requirement for SCARB1-dependent cholesterol acquisition *in vivo* (14).

Compared with LDLR-mediated uptake, HDL-related cholesterol uptake through SCARB1 may confer several potential metabolic advantages. First, because SCARB1 mediates selective CE transfer rather than bulk internalization of the entire lipoprotein particle, this route may help avoid abrupt short-term accumulation of FC and thereby reduce membrane stress and cholesterol-induced endoplasmic reticulum stress (5, 29–31, 39). Second, under the pseudo-hypoxic state associated with persistent HIF activation in ccRCC, selective uptake might theoretically reduce dependence on endo-lysosomal processing and lower metabolic burden (5, 10, 11). However, these proposed advantages remain inferred rather than directly demonstrated in ccRCC, because direct isotope-tracing or subcellular metabolic flux evidence comparing SCARB1- and LDLR-dependent cholesterol handling is still limited (10, 14, 15).

Taken together, current evidence supports the conclusion that SCARB1-mediated HDL-CE uptake is an important source of exogenous cholesterol in ccRCC, particularly in the context of relatively constrained endogenous cholesterol biosynthesis (3, 10, 14, 15, 28).

### 4.2 Relative functional insufficiency of cholesterol efflux mediated by the lxrα-ABCA1/ABCG1 axis

LXRα is a central transcriptional regulator of intracellular cholesterol homeostasis. Upon activation by oxysterols and related sterol ligands, LXRα induces the expression of downstream ATP-binding cassette transporters, especially ABCA1 and ABCG1, thereby promoting the efflux of excess intracellular free cholesterol (45, 46). More specifically, ABCA1 primarily mediates cholesterol and phospholipid transfer to lipid-poor ApoA-I, initiating nascent HDL formation, whereas ABCG1 mainly facilitates further cholesterol efflux to more mature HDL particles (39). Through this coordinated efflux program, the LXRα-ABCA1/ABCG1 axis plays an important role in limiting intracellular FC accumulation and reducing cholesterol-induced membrane perturbation and endoplasmic reticulum stress (39, 45, 47).

#### 4.2.1 Expression patterns of ABCA1 and ABCG1 in ccRCC

Multiple bioinformatic analyses based on TCGA KIRC and related datasets have demonstrated that the transcript abundance of ABCA1 and ABCG1 is elevated in ccRCC tissues compared with normal kidney tissues (2, 47, 48). Notably, this increased mRNA expression cannot be equated with enhanced functional cholesterol efflux capacity, and cell type-resolved single-cell RNA sequencing evidence further explains this contradictory phenomenon and requires cautious interpretation of bulk transcriptomic results.

Single-cell RNA sequencing studies suggest that the upregulated ABCA1 transcripts are predominantly derived from macrophages and stromal cell populations in the tumor microenvironment, rather than malignant renal epithelial cells, which exhibit negligible changes in ABCA1 expression (7, 49). By contrast, ABCG1 shows broader expression across both tumor cells and tumor-associated myeloid populations, including M2-like macrophages, implying that ABCG1 may be more directly involved than ABCA1 in tumor cell-intrinsic cholesterol handling in ccRCC (7, 49). In addition, several database-based survival analyses have reported that relatively higher expression of ABCA1 and ABCG1 is associated with a more favorable prognosis in ccRCC (2, 47, 48). However, these prognostic correlations are largely inferred from bulk RNA-seq data, which cannot distinguish transcript sources and are heavily influenced by the immune and stromal cellular composition of tumor tissues. Collectively, the elevated transcript abundance of ABCA1/ABCG1 does not represent enhanced functional activity of the cholesterol efflux axis, and the prognostic associations of the genes should be interpreted with caution, and their functional significance in tumor cells still requires further validation (7, 13, 49).

#### 4.2.2 Relative functional insufficiency of LXRα-ABCA1/ABCG1 axis mediated cholesterol efflux in ccRCC

A core regulatory characteristic of cholesterol metabolism in ccRCC is the functional insufficiency of the LXRα-ABCA1/ABCG1 cholesterol efflux axis, which occurs despite elevated ABCA1 and ABCG1 transcript levels in tumor tissues. LXRα forms a heterodimer with RXR and, upon activation by endogenous sterol ligands such as oxysterols, binds to LXR response elements in target promoters to induce the transcription of ABCA1 and ABCG1 (39, 45–47). Although ABCA1 and ABCG1 expression is increased in ccRCC, the cholesterol efflux capacity mediated by the LXRα-ABCA1/ABCG1 axis may still be relatively insufficient at the functional level. Several non-mutually exclusive mechanisms may account for this phenomenon (7, 45, 47, 50).

**FIGURE 3:**
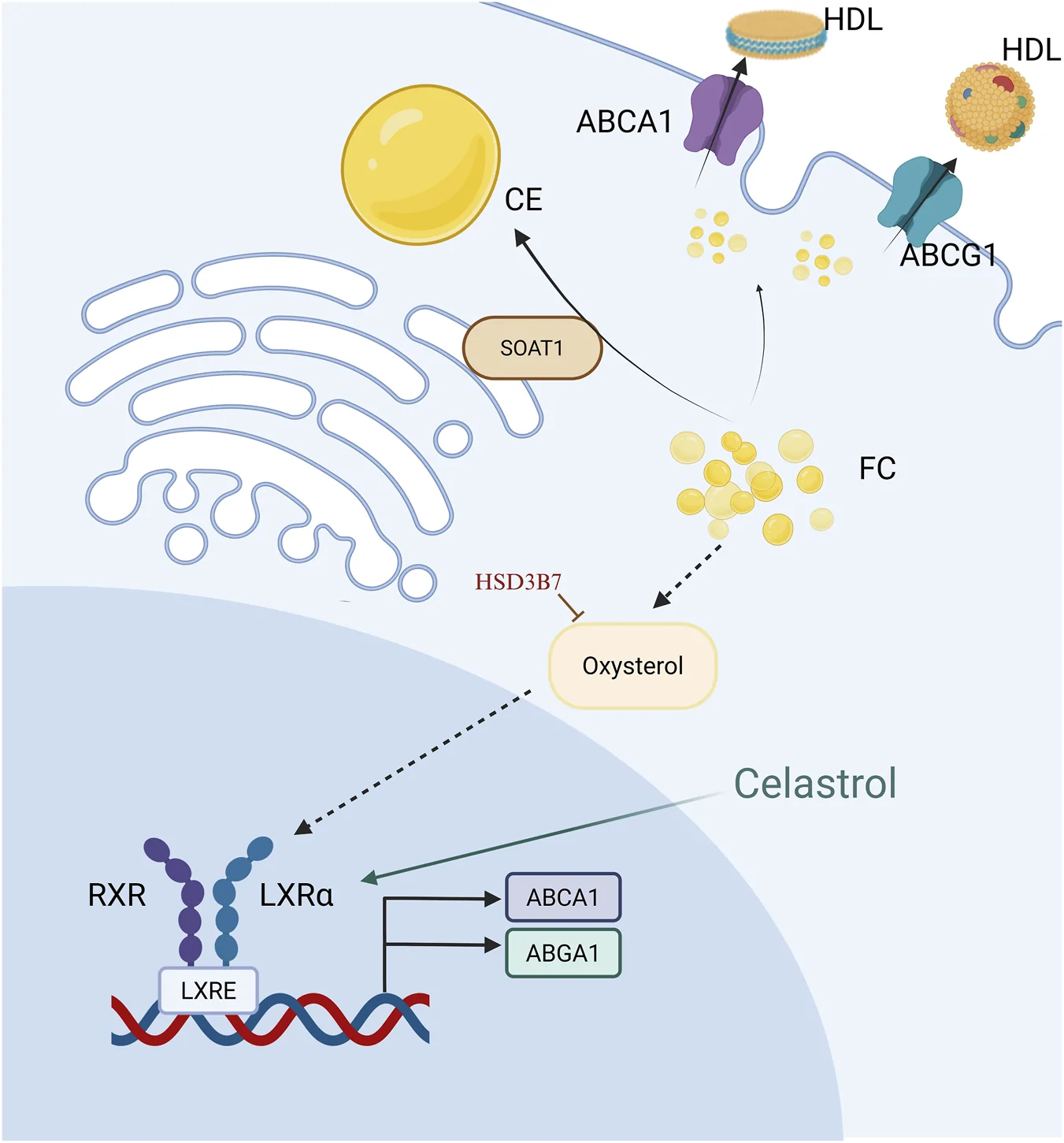
Proposed insufficiency of the LXRα–ABCA1/ABCG1 cholesterol efflux axis in ccRCC. Free cholesterol (FC) in ccRCC can be re-esterified by SOAT1 into cholesteryl esters, reducing the sterol pool that is available for oxysterol generation. Limited oxysterol availability and HSD3B7-associated metabolism, may weaken LXRα activation and downstream ABCA1/ABCG1-mediated cholesterol efflux. Celastrol is shown as a potential activator of the LXRα–ABCA1/ABCG1 axis. Created in BioRender. https://BioRender.com/x9ggc9x.

First, the bioavailability of endogenous LXR ligands, especially oxysterols, may be limited. In ccRCC, rapid SOAT1-mediated re-esterification channels excess FC into cholesteryl ester storage within lipid droplets, which may reduce the pool of free sterol available for conversion into signaling-active oxysterols and thereby blunt effective LXRα activation (5, 10, 14, 31, 39, 45). Second, HSD3B7, a key enzyme involved in oxysterol utilization/catabolism within bile acid biosynthetic metabolism, is upregulated in ccRCC, and recent work suggests that HSD3B7 activity helps prevent the accumulation of potentially toxic cholesterol-derived oxysterols; this raises the possibility that enhanced HSD3B7-dependent metabolism may further reduce endogenous LXR ligand availability (50) (Figure 3). Third, SCARB1-mediated selective CE uptake is highly efficient in ccRCC, and the rapid consumption of intracellular FC by re-esterification may help maintain a concentration gradient favoring continued cholesterol influx, thereby functionally weakening the net driving force for cholesterol efflux (10, 14–16).

Importantly, all the above mechanistic inferences are largely indirect. To date, there is still a critical lack of direct experimental evidence in ccRCC research, in particular, no direct measurements of intracellular cholesterol efflux rates in ccRCC malignant cells have been reported. In addition, systematic quantitative profiling of oxysterol species, direct measurement of cholesterol efflux flux, and compartment-resolved analyses linking SOAT1, HSD3B7, and LXRα activity are still needed (7, 10, 14, 45, 50).

#### 4.2.3 Potential therapeutic strategies for activating the LXRα-ABCA1/ABCG1 axis

Emerging evidence suggests that pharmacological activation of the LXRα-ABCA1/ABCG1 axis may partially restore cholesterol efflux capacity in ccRCC (2, 45, 51). Celastrol, a bioactive triterpenoid, has been reported to induce LXRα expression and increase the expression of the downstream cholesterol transporters ABCA1 and ABCG1 in ccRCC cell models, including 786-O and A498 cells (45). In these systems, Celastrol treatment was associated with enhanced cholesterol efflux, reduced intracellular lipid droplet accumulation, and induction of lipophagy, together with suppression of proliferation, migration, and epithelial-mesenchymal transition-related phenotypes (45). In xenograft models, Celastrol also showed dose-dependent inhibition of tumor growth (45).

These findings support the possibility that activation of the LXRα-dependent efflux program may, at least in part, counteract the lipid storage phenotype of ccRCC (2, 45, 51). Mechanistically, LXRα is a well-established transcriptional regulator of ABCA1 and ABCG1, both of which are central mediators of cellular cholesterol export (47). Accordingly, within the conceptual framework of “increased input-esterification buffering-insufficient efflux,” pharmacological activation of LXRα may represent a means to enhance cholesterol clearance and reduce dependence on SOAT1-mediated re-esterification and lipid droplet sequestration (2, 45, 51).

However, this interpretation should be made cautiously. Although Celastrol-induced activation of the LXRα-ABCA1/ABCG1 pathway has been demonstrated in pharmacological studies (45), whether basal cholesterol efflux is intrinsically and functionally “insufficient” in ccRCC remains to be directly established. At present, this view is supported mainly by indirect evidence, including the prominent lipid storage phenotype, the apparent reliance on exogenous cholesterol acquisition, and the therapeutic response to perturbation of lipid handling pathways, rather than by direct quantitative efflux or isotope-tracing measurements (2, 14, 45, 51). In addition, the extent to which LXRα agonists can achieve sustained and tumor-selective cholesterol efflux enhancement *in vivo*, without causing systemic lipid disturbance, remains unclear (51). Whether such agents can be rationally combined with HIF-2α inhibitors, VEGF-targeted therapies, or immune checkpoint blockade also requires further preclinical and clinical validation (52).

### 4.3 SOAT1-driven lipid Re-esterification process

In ccRCC cells, SCARB1-mediated selective uptake of HDL-derived CEs serves as an important source of exogenous cholesterol (7, 15). However, CEs are not directly transported into the lipid droplet after uptake. Studies have shown that SCARB1-mediated selective uptake of CEs can be delivered to membranous organelles, where they are converted into FC by hydrolases, the FC can then participate in membrane renewal and endoplasmic reticulum metabolism (15, 39).

The metabolic fates of FC mainly involve two aspects: on the one hand, FC can be used for the renewal of the plasma membrane and endomembrane system, supporting the supply of membrane components and the maintenance of membrane-associated signaling platforms under conditions of sustained cell proliferation, invasion, and metastasis (31, 39). On the other hand, excessive local accumulation of FC in the endoplasmic reticulum or membrane system will significantly increase the risk of endoplasmic reticulum stress and lipotoxicity (5, 29, 30). Therefore, a certain amount of FC is directed to the endoplasmic reticulum for re-esterification and sequestered in lipid droplets in the inert form of CEs, which is one of the important regulatory mechanisms by which cells adapt to lipid metabolic stress (5, 14, 15, 31).

In cell experiments, studies have also found that after blocking SCARB1 with BLT-1, FC levels only slightly decrease, while CE levels increase (15). A reasonable explanation is that lipid metabolism in ccRCC cells is not a single flow of uptake and efflux. In the context of insufficient efflux function, blocking SCARB1-mediated uptake with BLT-1 may stressfully reduce lipid efflux. To avoid membrane system pressure caused by FC accumulation, cells switch to activate downstream esterification buffering, rapidly converting highly active FC into relatively inert CEs for storage in lipid droplets, resulting in compensatory increases in CEs (5, 14, 15, 45, 47).

#### 4.3.1 SOAT1 as a central enzyme mediating FC re-esterification

SOAT1 is an ER-resident cholesterol esterification enzyme that catalyzes the conversion of FC into CE using fatty acyl-CoA as the acyl donor. It should be noted that older studies frequently referred to this enzyme as “ACAT-1,” whereas SOAT1 is the currently recommended gene name in contemporary nomenclature. In the context of ccRCC, the functional importance of SOAT1 likely extends beyond CE production *per se*, as it may also help buffer excess FC and thereby limit cholesterol-associated membrane stress and ER dysfunction (5, 10, 16).

Under conditions of increased cholesterol acquisition, SOAT1-mediated re-esterification is thought to provide a critical detoxifying mechanism by channeling FC into the relatively inert CE pool stored within lipid droplets (5, 16). Current evidence suggests that this process supports ER homeostasis and tumor cell fitness, whereas disruption of lipid storage programs can provoke ER stress and impair cell survival in ccRCC (5, 6). Consistent with this model, recent work has further linked cholesteryl ester accumulation to ccRCC progression, supporting the view that active cholesterol esterification is not merely a passive storage process but may contribute functionally to tumor maintenance (10, 16).

Additional evidence indicates that SOAT1 may be embedded within a broader lipid storage network in ccRCC. AUP1, an ER- and lipid droplet-associated protein implicated in neutral lipid accumulation, has been reported to promote lipid deposition in ccRCC cells (53). In that study, AUP1 knockdown reduced SOAT1 expression, whereas SOAT1 overexpression partially rescued the suppression of cell proliferation and the decrease in intracellular triglyceride and cholesterol-associated neutral lipid indices induced by AUP1 depletion (53). These findings suggest that SOAT1 may act as one downstream effector of AUP1-associated lipid storage, although the precise regulatory relationship between AUP1 and SOAT1, and whether this reflects direct transcriptional, post-transcriptional, or metabolic control, remains to be clarified (53).

#### 4.3.2 Potential auxiliary roles of the Aster/GRAMD1 machinery in supporting SOAT1-dependent cholesterol esterification

**FIGURE 4:**
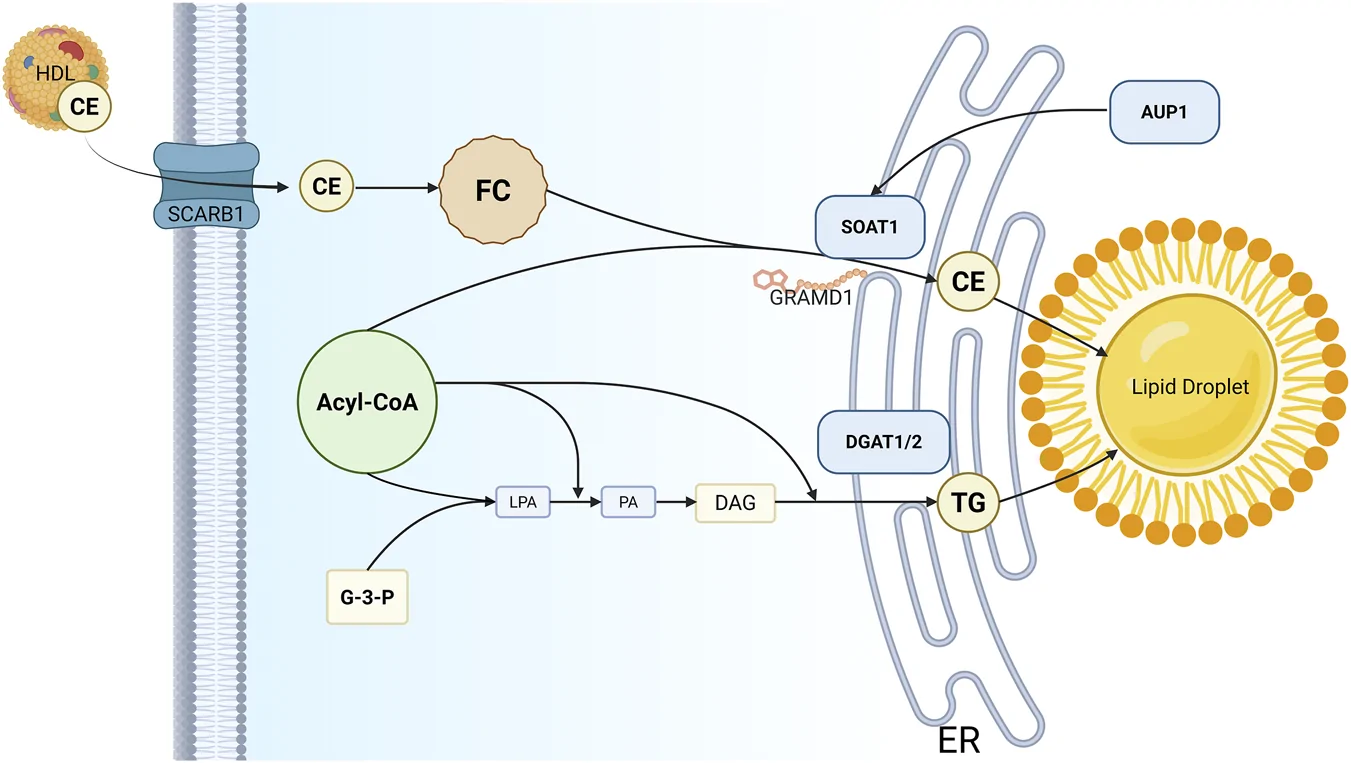
Coupling of the HDL-derived CE and fatty acid–TG pathways in ccRCC. HDL-derived CE is taken up through SCARB1, converted to FC, and re-esterified by SOAT1 in the ER for storage in lipid droplets. In parallel, fatty acyl-CoA supports TG synthesis through DGAT1/2. These pathways are coupled through shared fatty acyl-CoA during lipid droplet formation. GRAMD1 is shown as a proposed auxiliary mediator of cholesterol transport to the ER. Created in BioRender. https://BioRender.com/j1conyr.

In addition to SOAT1 expression itself, flux through the cholesterol esterification pathway is also likely to depend on the availability of FC within the ER. Members of the Aster/GRAMD1 family have been shown in other cellular systems to sense accessible cholesterol at plasma membrane-ER contact sites and mediate its non-vesicular transport to the ER (38, 39, 54, 55). In principle, this process could facilitate substrate delivery for SOAT1 and thereby support continued conversion of FC into CE (Figure 4).

From a mechanistic perspective, ongoing SOAT1-dependent esterification may help maintain relatively low ER FC availability, which could in turn favor continued cholesterol movement from membrane compartments to the ER along an effective concentration gradient (39, 54). This model raises the possibility that cholesterol transport, re-esterification, and lipid droplet sequestration may operate as a functionally coupled circuit that limits prolonged FC retention in membrane systems and mitigates cholesterol-associated membrane stress. However, this framework remains largely inferential in the context of ccRCC and has not yet been directly demonstrated by flux analysis or compartment-resolved tracing approaches.

Importantly, the currently available evidence for Aster/GRAMD1-mediated plasma membrane-to-ER cholesterol transfer derives primarily from non-ccRCC experimental systems (38, 39, 54, 55). Whether this transport module is active in ccRCC, whether it materially contributes to SOAT1-dependent CE accumulation, and whether it is regulated by VHL-HIF signaling or broader lipid metabolic rewiring remain unclear and require direct validation in tumor-relevant models (the genes that were related with ccRCC lipid metabolic reprogramming were listed in Table 1).

**TABLE 1:** Key lipid metabolic molecules and regulatory mechanisms involved in ccRCC lipid reprogramming.

Lipid regulator	Key molecules	Biological roles
Lipid sensing and transcriptional regulation	HIF-2α	VHL loss related regulator of tumor hypoxia adaptation and lipid remodeling
Lipid transcription regulation	SREBP1/SREBP2	HIF/SREBP-mediated lipid metabolic regulator
HDL related cholesteryl ester uptake and storage	SCARB1	VHL and HIF-2α related HDL derived cholesterol uptake
	SOAT1	Cholesterol esterification and lipid droplet formation
	GRAMD1 family	HDL associated intracellular cholesterol trafficking
Cholesterol efflux	ABCA1/ABCG1	Altered cholesterol efflux regulation
FA-TG axis	ACLY	Acetyl-CoA generation for FA synthesis
	FASN	SREBP1-associated lipogenic activation and fatty acid synthesis
	DGAT1/DGAT2	TG synthesis
FA oxidation pathway	CPT1A	HIF-associated mitochondrial fatty acid oxidation suppression

#### 4.3.3 Implications of lipid re-esterification for the tumor immune microenvironment

Because of excessive lipid accumulation, ccRCC cells face a substantial cholesterol burden. Excess FC can impair membrane homeostasis and aggravate ER stress, whereas suppression of fatty acid β-oxidation further limits the entry of accumulated lipids into energy metabolism (5, 11, 30, 31). Under this pressure, ccRCC appears to rely heavily on efficient esterification, rapidly converting FC into relatively inert CE, which are subsequently sequestered in the core of lipid droplets (5, 14, 16, 31). This esterification-sequestration strategy promotes lipid phase separation and helps buffer the toxic burden imposed by active lipid species on membrane systems (5, 30, 31).

However, when intracellular buffering capacity becomes insufficient, lipid dysregulation may extend beyond tumor cells and reshape the tumor microenvironment (TME). Recent lipidomic analyses of fresh ccRCC tissues and matched extracellular fluids showed enrichment of oleate-associated lipid species in the extracellular tumor space, with a pattern distinct from plasma, suggesting local tumor-associated metabolic remodeling rather than simple blood contamination (13). In the same study, the abundance of these extracellular oleate-related lipids was negatively correlated with CD8^+^ T cell infiltration, and oleate directly suppressed activation and perforin production of tumor-infiltrating CD8^+^ T cells *ex vivo* (13). Given that ccRCC is characterized by abundant but frequently dysfunctional/exhausted T cell infiltrates (49), these findings support the notion that a lipid-rich milieu contributes to local immune suppression.

Consistently, previous studies have shown that excessive cholesterol accumulation in CD8^+^ T cells can trigger persistent ER stress and promote expression of immune checkpoint molecules such as PD-1 and 2B4 through the IRE1α-XBP1 pathway (56, 57). In parallel, tumor-associated macrophages may also acquire immunosuppressive properties through enhanced uptake of oxidized or tumor-derived lipids, thereby further reinforcing suppression of antitumor immunity (7, 24, 58, 59). Taken together, current evidence suggests that lipid accumulation in ccRCC has consequences extending beyond tumor cell-intrinsic metabolism and exerts important effects on the TME (13, 49, 56, 57).

## 5 Cooperative regulation of lipid metabolism in ccRCC by classical fatty acid-TG pathway and the HDL-CE pathway

In ccRCC, characteristic loss of the VHL gene results in sustained activation of HIF-2α, which can influence both the classical fatty acid-TG metabolic axis and the HDL-CE metabolic pathway (5, 12, 15, 16, 34, 42). Lipidomic analyses have shown that, within ccRCC tumor tissues, CE and TG each account for approximately 50% of storage lipids, whereas CE is relatively less abundant in adjacent normal kidney tissue (4, 13, 36). These findings indicate that the classical fatty acid-TG pathway and the HDL-CE pathway jointly contribute to lipid droplet homeostasis in ccRCC (4, 13, 34).

The interaction between these two metabolic axes is not limited to the concomitant deposition of TG and CE. A key point is that they share the common substrate fatty acyl-CoA. DGAT1/2 use fatty acyl-CoA to convert diacylglycerol into TG, whereas SOAT1 depends on long-chain fatty acyl-CoA to re-esterify FC into CE (31–33, 60). These two pathways converge functionally at the ER-lipid droplet interface. TG synthesis mainly contributes to the bulk neutral-lipid substrate required for lipid droplet growth and helps buffer excess fatty acids, whereas SOAT1-mediated cholesterol esterification, under conditions of high cholesterol influx, converts chemically active FC into relatively inert CE and promotes its sequestration in the lipid droplet core, thereby reducing membrane lipid stress and the risk of ER dysfunction (5, 16, 31, 33).

Because both pathways rely on shared acyl-CoA pools and feed neutral lipids into lipid droplets, inhibition of one branch may increase selective pressure on the other and create the potential for compensatory rerouting of lipid flux (31–33). Thus, coordinated regulation of lipid homeostasis by DGAT and SOAT1 may help explain the distinctive TG-CE composition of lipid droplets in ccRCC and provides a mechanistic rationale for considering combined blockade of compensatory storage pathways as a strategy to enhance therapeutic efficacy (5, 16, 32, 34).

## 6 Clinical relevance of lipid metabolism in ccRCC and potential therapeutic targets

### 6.1 SCARB1 inhibitors

SCARB1 mediated selective uptake of HDL derived cholesteryl ester is a critical exogenous cholesterol acquisition pathway for ccRCC, rendering SCARB1 a promising therapeutic vulnerability for the cancer (14, 15). In preclinical *in vitro* studies and mouse xenograft models, genetic or pharmacological inhibition of SCARB1, including the use of the small-molecule inhibitor BLT-1, has been shown to reduce HDL uptake, increase reactive oxygen species, attenuate PI3K/AKT signaling, induce cell-cycle arrest and apoptosis, and suppress tumor growth (14, 15). These findings confirm the cholesterol dependence of ccRCC cells and support the therapeutic potential of SCARB1 targeted strategies (10, 14).

Clinically, SCARB1 expression has been reported to be related with patients TG level, and the SCARB1 antagonist ITX5061 has previously been evaluated in human studies in the context of hepatitis C virus infection, based on its ability to block SCARB1-dependent viral entry (60, 61). Although its application in renal tumors remains to be validated, this prior clinical experience shall provide useful information regarding drug exposure and tolerability in humans (60, 61).

### 6.2 SOAT1 inhibitors

Given the proposed role of SOAT1 in buffering excess FC through re-esterification, pharmacological inhibition of SOAT1 represents another rational strategy to disrupt lipid homeostasis in ccRCC (5, 10, 16). In principle, blocking SOAT1 is expected to reduce cholesteryl ester storage, increase intracellular FC burden, and compromise tumor cell fitness by enhancing cholesterol-associated stress (5, 16). This strategy is particularly attractive within the framework of “increased input-esterification buffering-insufficient efflux,” as it would target the buffering arm that allows ccRCC cells to tolerate sustained cholesterol influx (5, 10).

Several clinical experience with SOAT1-targeting agents is already available in a few cancer types, for instance, Nevanimibe (ATR-101), a SOAT1 inhibitor, has completed a phase I study in adrenocortical carcinoma, providing pharmacokinetic and tolerability data relevant to clinical translation (62). In addition, mitotane, an approved drug for adrenocortical carcinoma, has been suggested to exert part of its cytotoxic effect through SOAT1 inhibition, leading to lipid-mediated ER stress and cell death (63). Nevertheless, these findings are outside of renal tumors, their applicability in ccRCC require further systematic preclinical and clinical validation.

### 6.3 LXRα-ABCA1/ABCG1 agonists or activators

Contrary to the inhibitory strategies targeting cholesterol influx and esterification, pharmacological activation of the LXRα-ABCA1/ABCG1 axis represents a conceptually complementary strategy that enhances cholesterol efflux rather than suppress influx or esterification (45, 47). In preclinical ccRCC models, pharmacological activation of LXR signaling was associated with reduced lipid accumulation and impaired tumor growth (45). In particular, Celastrol has been reported to induce LXRα expression, increase ABCA1 and ABCG1 expression, promote cholesterol efflux, reduce intracellular lipid droplet content, induce lipophagy, and suppress proliferation, migration, and epithelial-mesenchymal transition-related phenotypes in ccRCC cell lines (45). In xenograft models, Celastrol also inhibited tumor growth in a dose-dependent manner (45).

While these preclinical findings support the tumor-suppressive potential of LXR agonism, the clinical translation of this strategy faces notable limitations and safety concerns. Existing evidence is confined to single preclinical pharmacological models and lacks validation across diverse ccRCC molecular subtypes (45). Moreover, systemic LXR activation induces lipogenic side effects and central nervous system toxicity, as evidenced by the terminated clinical development of the LXR agonist LXR-623 (64, 65). Future clinical application relies on the development of tumor-selective LXR ligands, targeted delivery systems, or combination regimens to minimize systemic toxicity while retaining metabolic antitumor activity (64, 65).

### 6.4 DGAT1/2 and upstream lipogenic nodes inhibitors

Triglyceride synthesis which is catalyzed by DGAT1/2, is also of potential relevance in ccRCC because DGAT-dependent triglyceride storage and SOAT1-dependent cholesterol esterification are biochemically linked through their shared use of fatty acyl-CoA (31, 32, 34). This raises the possibility of substrate competition and compensatory flux redistribution between the TG and CE arms of lipid droplet formation. As a result, inhibition of one branch alone may be partially offset by increased flux through the other, providing a rationale for considering coordinated or combination targeting strategies (31, 32, 34).

DGAT inhibitors have mature clinical development experience in metabolic diseases, though not in ccRCC. The DGAT2 inhibitor ervogastat has entered clinical evaluation in metabolic liver disease, including phase 2 testing in patients with MASH (66). The DGAT1 inhibitor pradigastat has also been clinically investigated in rare disorders such as familial chylomicronemia syndrome (67). These studies provide useful information on feasibility, exposure, and pharmacokinetic references (66, 67). Preclinical studies have confirmed the oncogenic JMJD6-DGAT1 axis in ccRCC, but whether DGAT inhibition can block tumor lipid buffering or trigger compensatory cholesterol esterification *in vivo* remains unvalidated.

In addition to DGAT1/2, upstream lipogenic enzymes such as ACLY and FASN may also have translational relevance in ccRCC (68, 69). Bempedoic acid, an ATP-citrate lyase (ACLY) inhibitor, is already approved in the United States to reduce the risk of myocardial infarction and coronary revascularization in appropriate cardiovascular populations, illustrating that ACLY can be targeted in humans within a clinically tractable safety framework (69).

Likewise, the FASN inhibitor denifanstat (TVB-2640) has completed early-phase clinical evaluation in advanced solid tumors, with ongoing trials in oncology and metabolic disease (70). In ccRCC, these prior translational experiences may help lower the barrier for repurposing or adapting lipid metabolism-directed interventions. Even so, the antitumor efficacy of ACLY or FASN inhibition in ccRCC remains insufficiently defined, and whether these agents should be used alone or combined with HIF-2α inhibitors, VEGF-targeted therapy, or immunotherapy requires further study (27, 60).

Although most of aforementioned lipid metabolic targeted strategies were based on *in vitro* studies and mouse xenograft models, more systematic and rigorous clinical validation are still needed, the propose of predictive biomarkers is critical to improve treatment strategies, screen benefiting patient subgroups, and enhance the metabolic therapy in ccRCC (all potential gene targets and therapeutic strategies, as well as the current evidence regarding the clinical application of these genes were listed in Table 2).

**TABLE 2:** Potential therapeutic strategies targeting lipid metabolic regulators.

Gene targets	Therapeutic strategies	Current evidence
HIF-2α	Inhibition of hypoxia-driven cancer metabolism	Clinically validated gene target in ccRCC
SCARB1	Inhibition of HDL-cholesterol uptake	*in vitro* studies and mouse xenograft models were reported, human experiments still lacking
SOAT1	Inhibition of cholesterol esterification	SOAT1-targeting agents were reported in other cancers, not ccRCC yet
LXRα- ABCA1/ABCG1	Activation of cholesterol efflux pathways	*in vitro* studies and mouse xenograft models were reported, more experience are needed before clinical application
SREBP1, ACLY, FASN	Targeting lipid synthesis pathways	SREBP1-targeting agents were reported in other cancers, antitumor efficacy of ACLY or FASN inhibition still lacking
DGAT1/2	Inhibition of lipid storage	DGAT1/2 inhibitors were reported in other diseases, not ccRCC yet
CPT1A	Modulation of fatty acid oxidation metabolism	*in vitro* studies and mouse xenograft models were reported, human experiments still lacking

## 7 Current limitations and future directions

As discussed above, accumulating evidence suggests that, in addition to the classical FA-TG axis, the HDL-cholesterol pathway also contributes to lipid metabolic reprogramming in ccRCC (10, 13, 14, 16). However, the current evidence base remains incomplete. Much of the existing literature is still centered on expression profiling, association analyses, and *in vitro* perturbation studies, whereas direct evidence for transcriptional regulation, subcellular sterol trafficking, intercellular lipid exchange within the tumor microenvironment, and clinically actionable metabolic dependencies remains limited (2, 10, 51). As a result, several key mechanistic and translational questions remain unresolved.

### 7.1 The direct regulatory mechanism linking HIF-2α to SCARB1 remains unclear

Current evidence supports a functional association between VHL loss, sustained HIF-2α activation, and increased SCARB1 expression in ccRCC (10, 15, 16, 42). This relationship is biologically plausible within the broader context of VHL-HIF-driven metabolic rewiring, and SCARB1-dependent HDL-cholesteryl ester uptake has been shown to contribute to lipid accumulation and tumor-supportive phenotypes in ccRCC models (7, 10, 15). Nevertheless, whether SCARB1 is a direct transcriptional target of HIF-2α has not yet been established. In particular, direct chromatin-level evidence—such as HIF-2α ChIP-seq or ChIP-qPCR occupancy at the SCARB1 locus, identification of a functional hypoxia-responsive element, or promoter-reporter validation after site-directed mutation—has not yet been reported.

A similar limitation applies to the proposed involvement of SREBP in SCARB1 regulation. Earlier studies demonstrated that SREBP-1a can bind sterol-responsive cis elements in the rat SCARB1 promoter and activate promoter activity *in vitro* (43). Although these findings remain mechanistically informative, they were generated primarily in non-ccRCC systems using promoter analysis and electrophoretic mobility shift assays rather than endogenous chromatin-based approaches (43). Thus, by current standards, they do not by themselves establish direct transcriptional control of SCARB1 under physiological chromatin conditions in ccRCC. More recent studies linking HIF-2α to SREBP-dependent lipogenic programs in ccRCC further support the possibility of indirect regulatory coupling (12), but direct evidence that SREBP occupies and regulates the endogenous SCARB1 locus in ccRCC is still lacking. Future work integrating ChIP-seq, CUT&RUN/CUT&Tag, CRISPR-based enhancer perturbation, and transcriptional flux analysis will be important to determine whether SCARB1 is regulated directly by HIF-2α, indirectly through SREBP-associated circuitry, or through a more complex multi-layered network.

### 7.2 The intracellular hydrolytic routing of HDL-derived cholesterol remains unresolved

Another major unresolved issue concerns the intracellular fate of HDL-derived cholesteryl ester after SCARB1-mediated uptake in ccRCC. In non-renal systems, SCARB1-delivered HDL-CE has been shown to undergo rapid hydrolysis through a neutral cholesteryl ester hydrolase pathway rather than the classical lysosomal route used for LDL-derived CE (71). In addition, studies in other cellular contexts suggest that accessible cholesterol can be transferred from the plasma membrane to the ER through Aster/GRAMD1-mediated non-vesicular transport (38, 39, 54, 55). Together, these observations provide a conceptual framework in which HDL-derived cholesterol could be hydrolyzed, redistributed across membrane compartments, and ultimately supplied to the ER for SOAT1-dependent re-esterification.

However, none of these routing steps has been quantitatively established in ccRCC. It remains unclear where HDL-derived CE is predominantly hydrolyzed after uptake, which hydrolase activities are rate-limiting, whether the resulting free cholesterol is preferentially routed toward membrane remodeling, signaling, mitochondrial metabolism, or re-esterification, and to what extent Aster/GRAMD1 family members participate in this process in tumor cells (38, 39, 54, 55, 71). Likewise, the relative contribution of lysosomal versus non-lysosomal handling, and the extent to which HDL-derived cholesterol enters the same metabolic pools as de novo synthesized or LDL-derived cholesterol, remain unknown. These gaps are particularly important because the proposed model of “increased input-esterification buffering-insufficient efflux” implicitly depends not only on uptake, but also on efficient intracellular routing from HDL-derived CE to free cholesterol and then into the SOAT1-lipid droplet buffering arm.

Addressing these questions will require approaches that go beyond steady-state expression analysis. Future studies should incorporate isotope-resolved metabolic tracing of HDL-derived cholesterol, compartment-resolved sterol imaging, spatial lipidomics, subcellular fractionation coupled to lipidomics, and perturbation of candidate transport and hydrolase nodes in tumor-relevant systems. Such work will be necessary to determine whether HDL-derived cholesterol in ccRCC is primarily used for membrane adaptation, stored after re-esterification, released into the extracellular tumor milieu, or dynamically redistributed among these fates.

### 7.3 Noncoding RNA-mediated regulation of SCARB1 associated with the HDL-CE axis is an emerging field

Recent studies suggest that noncoding RNAs may participate in regulation of the SCARB1/HDL-CE pathway in ccRCC (7, 44). In particular, circABCA1 has been reported to be upregulated in ccRCC and to promote cholesterol metabolic reprogramming through an IGF2BP3-dependent mechanism that stabilizes SCARB1 mRNA (7). In that study, circABCA1 was associated with enhanced selective uptake of HDL-derived cholesteryl ester and with a shift toward a cholesterol-retentive phenotype, while also promoting tumor progression and M2 macrophage polarization (7). Likewise, circ_0003146 has been shown to increase SCARB1 expression by functioning as a sponge for miR-1272, thereby facilitating malignant phenotypes in ccRCC cells (44). Together, these findings raise the possibility that post-transcriptional regulation of SCARB1 by noncoding RNAs may contribute to sustained cholesterol influx in at least a subset of ccRCC tumors (7, 44).

However, this field remains at an early stage. Available studies are still limited in number, and current evidence derives mainly from individual circRNA-centered investigations rather than from systematic mapping of the broader noncoding RNA regulatory landscape (7, 44). In addition, whether these RNA-mediated effects operate independently of the VHL-HIF program, cooperate with HIF-2α- or SREBP-associated transcriptional circuitry, or vary across molecular subtypes of ccRCC remains unclear. More broadly, although these findings provide a useful framework for considering noncoding RNA control of the SCARB1/HDL-CE axis, further validation in larger patient cohorts and mechanistic models will be required before firm conclusions can be drawn regarding their generality or therapeutic relevance.

### 7.4 Validation of Aster/GRAMD1 support for SOAT1-dependent cholesterol esterification in ccRCC

As discussed above, existing evidence that members of the Aster/GRAMD1 family mediate transport of accessible cholesterol from the plasma membrane to the ER is derived mainly from non-ccRCC cellular systems (38, 39, 54, 55). These studies support a model in which cholesterol transfer at membrane contact sites may help maintain ER sterol supply and thereby facilitate downstream esterification. In the context of ccRCC, this raises the possibility that Aster/GRAMD1 proteins may support high-flux SOAT1-dependent re-esterification under conditions of increased cholesterol influx. However, whether this transport module is functionally required in ccRCC, whether it becomes rate-limiting when SCARB1-mediated cholesterol uptake is high, and whether it materially contributes to cholesteryl ester accumulation in lipid droplets remain unknown (38, 39, 54, 55).

At present, direct support from tumor-relevant genetic interaction studies, compartment-specific cholesterol quantification, and CE flux measurements is lacking. Accordingly, the proposed role of Aster/GRAMD1 in sustaining the “increased input-esterification buffering” arm of the ccRCC lipid program should still be regarded as mechanistically plausible rather than experimentally established. Future studies should therefore examine ER free cholesterol pools, CE generation rates, lipid droplet CE content, and stress-related phenotypes following genetic perturbation of GRAMD1 family members under conditions of elevated SCARB1-dependent cholesterol uptake. Parallel analyses combined with SOAT1 inhibition would be particularly informative for determining whether Aster/GRAMD1 acts upstream of, in parallel with, or only contingently alongside the SOAT1 buffering pathway in ccRCC.

## 8 Discussion

In summary, this review first outlines the basic pathways of lipid metabolism in ccRCC, with particular emphasis on HDL-related cholesterol influx, cholesterol efflux, and SOAT1-mediated re-esterification. We further discuss how these mechanisms together establish an HDL-CE-centered metabolic route and how this route cooperates with the classical fatty acid-TG pathway during lipid droplet accumulation. Finally, we summarize potential therapeutic targets within these pathways and highlight several key evidence gaps that remain in the field. A more detailed understanding of these issues will improve our insight into the mechanisms underlying the characteristic clear cell phenotype of ccRCC and may help identify new metabolic vulnerabilities for therapeutic intervention.
